# Organised and opportunistic prevention in primary health care: estimation of missed opportunities by population based health interview surveys in Hungary

**DOI:** 10.1186/s12875-020-01200-2

**Published:** 2020-06-24

**Authors:** János Sándor, Ildikó Tokaji, Nouh Harsha, Magor Papp, Róza Ádány, Árpád Czifra

**Affiliations:** 1 Department of Preventive Medicine, Faculty of Public Health, University of Debrecen, Kassai26, Debrecen, 4026 Hungary; 2 Department of Life Quality, Hungarian Central Statistical Office, Keleti Károly 5–7, Budapest, 1024 Hungary

**Keywords:** Organised prevention, Opportunistic prevention, Primary care, Hypertension screening, Diabetes mellitus screening, Influenza vaccination

## Abstract

**Background:**

Improvement of preventive services for adults can be achieved by opportunistic or organised methods in primary care. The unexploited opportunities of these approaches were estimated by our investigation.

**Methods:**

Data from the Hungarian implementation of European Health Interview Surveys in 2009 (*N* = 4709) and 2014 (*N* = 5352) were analysed. Proportion of subjects used interventions in target group (screening for hypertension and diabetes mellitus, and influenza vaccination) within a year were calculated. Taking into consideration recommendations for the frequency of intervention, numbers of missed interventions among patients visited a general practitioner in a year and among patients did not visit a general practitioner in a year were calculated in order to describe missed opportunities that could be utilised by opportunistic or organised approaches. Numbers of missed interventions were estimated for the entire population of the country and for an average-sized general medical practice.

**Results:**

Implementation ratio were 66.8% for blood pressure measurement among subjects above 40 years and free of diagnosed hypertension; 63.5% for checking blood glucose among adults above 45 and overweighed and free of diagnosed diabetes mellitus; and 19.1% for vaccination against seasonal influenza. There were 4.1 million interventions implemented a year in Hungary, most of the (3.8 million) among adults visited general practitioner in a year. The number of missed interventions was 4.5 million a year; mostly (3.4 million) among persons visited general practitioner in a year. For Hungary, the opportunistic and organised missed opportunities were estimated to be 561,098, and 1,150,321 for hypertension screening; 363,270, and 227,543 for diabetes mellitus screening; 2,784,072, and 380,033 for influenza vaccination among the < 60 years old high risk subjects, and 3,029,700 and 494,150 for influenza vaccination among more than 60 years old adults, respectively. By implementing all missed services, the workload in an average-sized general medical practice would be increased by 12–13 opportunistic and 4–5 organised interventions a week.

**Conclusions:**

The studied interventions are much less used than recommended. The opportunistic missed opportunities is prevailing for influenza vaccination, and the organised one is for hypertension screening. The two approaches have similar significance for diabetes mellitus screening.

## Background

Despite some uncertainties about the scientific basis of the Declaration of Alma-Ata [1], evidence showing that public health problems can be reduced effectively through primary health care (PHC) has become convincing over the last four decades [2]. Although the original concept proposed by the Declaration is being adapted to the changing world, the provision for preventive services by PHC professionals has remained constant [3–5].

The feasibility of solving public health problems through the preventive services of PHC was convincingly demonstrated in the 1990s [6, 7]. Organisations have been established to develop recommendations for preventive interventions at the PHC level [8, 9].

The growing body of evidence-supported PHC interventions has established effective PHC-level prevention delivery for individuals visiting a general practitioner (GP) or GP’s co-workers. Regarding practice, the higher the ability of the PHC structure to deliver these services, the higher the health gain for the population served. Several PHC models have been developed to improve the effectiveness of PHC in this respect [10]. Development of human capacities, improvement in resources, support by monitoring, operating pay for performance systems, application of provider reminders, etc., can contribute to more effective opportunistic preventive practices in PHC delivery [11–13].

One of the most important limitations of the opportunistic approaches with respect to the public health impact is the lack of influence on the part of the population that does not have contact with PHC professionals. Obviously, the higher the reached proportion of the target population, the more effective the organised intervention. Considering the expertise needed for organised prevention services, a widening of professional base is required. It has to be accompanied with the adaption of guidelines for the PHC setting for health professionals such as physiotherapists, dieticians, and psychologists, elaboration of recruitment strategies, and monitoring of effectiveness [14].

There are many debates around the development of PHC and around modifications that enable the increased effectiveness of PHC preventive services [15–17]. If an opportunistic approach can ensure remarkably high effectiveness, then the added value of organised approaches is limited, and its cost-benefit ratio can be unfavourable. Conversely, if an opportunistic intervention has low performance, then the potential gains and cost-benefit relationships could be favourable for organised approaches. Therefore, in elaborating scenarios for PHC-level prevention development, it is necessary to estimate the gains that can be achieved by enforced opportunistic approaches and by enforced organised (population level call-based and regular health check-based) approaches.

Hungary had the second highest preventable mortality among member states of the European Union according to the latest report of the statistical office of the European Union (Eurostat) from 2015 (418/100,000). The number of excess preventable deaths in Hungary compared with the EU average (216/100,000) and the weighted average of Czech Republic, Slovakia, and Poland in a year is 11,788 and 7693, respectively. The role of missed PHC preventive services has not been quantified yet, but the delivery of PHC preventive services is far from the recommended [18–21]. Altogether, the prevention opportunities provided by PHC are among the important etiologic factors of preventable mortality.

Hungary participated in the first and second waves of the European Health Interview Surveys in 2009 and 2014 (EHIS2009, EHIS2014) [22, 23]. These population based, nationally representative surveys collected data on the use of some preventive services, lifestyle and chronic disorders allowing for the determination of target groups for preventive services. The use of different services of primary, secondary, and tertiary health care was also covered in the data collection. The questionnaire contained questions on the participation in screening for hypertension, diabetes mellitus, hypercholesterolemia, and for cervix, breast, and colorectal cancer, and on the vaccination against influenza. These surveys provide an opportunity for investigating the use of PHC-level preventive services delivered by GPs and its determinants only in case of the screening for hypertension, and diabetes mellitus, and vaccination against influenza, because (1) the cancer screening is organised without remarkable contribution of PHC in Hungary, and (2) the target group of hypercholesterolemia was not properly identifiable by the survey variables.

### Objectives

Our study aimed to achieve the following: (1) describe the proportion of users of preventive services, such as screening for hypertension and diabetes mellitus and vaccination against influenza, in target populations in Hungary; (2) describe how these proportions changed between 2009 and 2014; (3) determine what these proportions were among adults who did and did not visit a GP in a year to estimate the increase of the adherence to recommendations can be achieved by better opportunistic and better organised approaches; and (4) determine the impact of improved organised and opportunistic interventions on the workload of GPs.

## Methods

### Source of data

This investigation used anonymised individual records from EHIS2009 [24] and EHIS2014 [25]. These surveys were proposed by the European Commission and supervised by Eurostat [22].

According to the Eurostat defined design, multistage cluster sampling was applied in both surveys. First, the regions and settlement types, followed by age and sex, were taken into consideration in the random selection of representative subjects who were non-institutionalised (living not in collective households and institutions), aged 15 or above and residing in Hungary. The planned samples included 7000 and 9431 individuals in the first and second surveys, respectively. Surveys based on personal interviews were conducted by trained interviewers. Participants’ self-reported answers were recorded [26, 27].

The number of individuals participating in the surveys was 5051 (response rate 72%) and 5826 (response rate 62%) for the 2009 and 2014 surveys, respectively. Our investigation utilised only the records of subjects with age ≥ 20 years containing data on demographics, level of education, and lifestyle. The analysed database contained 4709 records from the first survey (67% of the sampled subjects with age ≥ 20 years produced useful records) and 5352 records from the second survey (effective response rate of 57%).

### Target groups for preventive interventions

Recommendations by the U.S. Preventive Services Task Force (USPSTF), American Diabetes Association (ADA), and the National Health Service in England (NHS) relevant for the period from 2009 to 2014 were used to define the target groups.

Screening for hypertension was recommended by the USPSTF for each adult at the PHC level [28]. There was no definitive recommendation for screening intervals, but it was known that yearly blood pressure checking can significantly increase the registered incidence of hypertension [29]. The Hungarian recommendation supports screening annually for persons above 40 years of age [30].

The ADA recommended screening for diabetes mellitus among those overweight and older than 45 years [31] without specifying the screening interval [32]. The Hungarian guidelines recommended screening every 3 years [33].

The NHS recommends annual vaccination against influenza in all adults above 65 years of age and for persons with high-risk chronic diseases less than 65 years old [34]. The Hungarian recommendation is similar to the NHS approach, but the threshold age is 60 years in Hungary [35].

The target groups for preventive interventions have been defined based on the above-mentioned recommendations and the availability of required data. Hypertension screening was investigated among persons aged at least 40 years old without known hypertension. Persons older than 45 years and overweight were considered targets for diabetes mellitus screening. Two target groups were studied for influenza vaccination: all persons at least 60 years old (≥60) and persons 20–59 years old with one of the high-risk diseases (< 60 HR). (High-risk diseases used are listed in **Appendix 1.**)

### Variables

The self-reported time of the last visit to a GP and the last hypertension screening, diabetes mellitus screening, and vaccination against influenza were recorded. Subjects were classified according to who had visited a GP in the previous year (in the previous 12 months) and who had visited a GP more than a year prior (longer than 12 months ago). On the other hand, adults who used these services within the past year (in the previous 12 months) were distinguished from subjects who did not use the services in the previous 12 months.

Participants’ socio-demographic status was described by sex (female; male), age (by 5-year age groups; 20–24, 85+), family status (single; married - living together; married - living separated; divorced; widow), and level of education (primary; vocational; high-school; tertiary). Lifestyle was described by alcohol consumption (occasional; never; moderate; regular; heavy) [36], BMI (thin; normal; overweighed; obese) [37], and smoking habit (ceased; never; moderate; heavy; using substance other than cigarettes). Among regular smokers, heavy (at least 20 cigarettes a day) and moderate smoking (less than 20 cigarettes a day) were distinguished [38]. Self-rated general health status (very bad; bad; fair; good; very good) and self-reported presence or absence of any chronic disease prevalence were detected. The self-reported chronic disease(s) were specified by the participants (the applied disease groups’ categories are listed in **Appendix 2**). The year of survey participation was also registered to differentiate subjects between EHIS2014 and EHIS2009.

### Statistical analysis

The number interventions and the proportion of persons used interventions within the previous year were calculated for each target group. On the basis of the observed proportions in the sample and the participants’ sampling weights (which were calculated considering the multistage sampling process and response rates), the number of interventions was estimated for the entire target population of Hungary. The following indicators were also determined:

- the number of persons who did not use a PHC service in the previous year; and the number of missed interventions for the previous year taking into consideration the recommended screening intervals (number of potential subjects available for preventive intervention by GPs, SAPI);
- by the distinction between subjects who visited a GP in the previous year and those who did not, the missed opportunities had been subcategorised by distinguishing the number of potential subjects available for opportunistic intervention by GPs (opportunistic SAPI), and the number of potential subjects who could be available for prevention services via an organised, call based screening and vaccination programme (organised SAPI);
- the number of interventions implemented by another physician not by a GP (non-GP) was estimated by the number of implemented interventions among adults who did not visit a GP in a year; (Taking into consideration that there can be adults who used preventive services and visited a GP in a year but their preventive interventions were carried out by a non-GP, this estimation is a lower approximation.)
- the number of implemented and missed interventions, and the number of potential opportunistic and organised interventions were calculated for an average-sized Hungarian general medical practice (GMP) by dividing the whole population estimated number of cases by the number of the GMPs in the country. (The number of GMPs providing care for adults was 5185 and 5099 in 2009 and 2014, respectively.)

Logistic regression models controlled for socio-demographic factors, lifestyle status, and health status were applied to determine the role of the GP visiting frequency on using preventive services according to the recommendations. The results were presented as the odds ratios (OR) with the corresponding 95% confidence intervals (95% CI). Regarding the year of the survey and the timing of the GP visit in the models, the reference categories were the year of the first survey in 2009 and a GP visit within a year of the survey.

## Results

### Observed proportion of implementation

In the target populations, the proportion of subjects who used PHC preventive services in the previous year were 66.8% for hypertension screening, 63.5% for diabetes mellitus screening, and 19.1% for vaccination against influenza (9.9% among the < 60 HR group and 25.8% among the ≥60 group) (Table 1).

**Table 1:** Proportion of subjects used preventive interventions per year in Hungary in different target groups (number of interventions / size of target group) as a function of self-reported timing of visiting a GP according to the European Health Interview Surveys from 2009 and 2014

	visiting GP	EHIS2009	EHIS2014	EHIS2009–2014
blood pressure measurement, 40+ years	within a year	83.2% (911/1095)	85.0% (1085/1276)	84.2% (1996/2371)
	more than a year	22.8% (104/456)	28.4% (156/550)	25.8% (260/1006)
	missing	100% (1/1)	0.0% (0/1)	50.0% (1/2)
	total	65.5% (1016/1552)	67.9% (1241/1827)	66.8% (2257/3379)
checking blood glucose, 45+ years, overweighed	within a year	69.4% (885/1276)	76.2% (1060/1391)	72.9% (1945/2667)
	more than a year	14.8% (33/223)	18.6% (58/312)	17.0% (91/535)
	missing	--- (0/0)	0.0% (0/2)	0.0% (0/2)
	total	61.2% (918/1499)	65.6% (1118/1705)	63.5% (2036/3204)
influenza vaccination, < 60 years, with risk factor	within a year	11.5% (123/1073)	9.2% (89/969)	10.4% (212/2042)
	more than a year	3.5% (5/143)	9.4% (10/106)	6.0% (15/249)
	missing	--- (0/0)	0.0% (0/1)	0.0% (0/1)
	total	10.5% (128/1216)	9.2% (99/1076)	9.9% (227/2292)
influenza vaccination, 60+ years	within a year	32.0% (413/1291)	24.6% (371/1509)	28.0% (784/2800)
	more than a year	8.1% (12/149)	9.7% (20/207)	9.0% (32/356)
	missing	--- (0/0)	0.0% (0/1)	0.0% (0/1)
	total	29.5% (425/1440)	22.8% (391/1717)	25.8% (816/3157)
together	within a year	49.3% (2332/4735)	50.6% (2605/5145)	50% (4937/9880)
	more than a year	15.9% (154/971)	20.8% (244/1175)	18.5% (398/2146)
	missing	100% (1/1)	0% (0/5)	16.7% (1/6)
	total	43.6% (2487/5707)	45% (2849/6325)	44.3% (5336/12032)

The differences between proportions of intervention among persons who visited a GP in the prior year and who did not were large. This difference was 84.2% vs 25.8, 72.9% vs 17.0, 10.4% vs 6.0, and 28.0% vs 9.0% for hypertension screening, diabetes mellitus screening, influenza vaccination among the < 60 HR group, and influenza vaccination among the ≥60 group, respectively (Table 1). The dominant role of the GP visit frequency has been confirmed by multivariate regression models. There was a strong association between the frequency of visiting a GP and the implementation of preventive actions in the target groups. According to the multivariate logistic regression models, the use of preventive services were much less frequent among subjects who did not visit a GP within a year: OR_screening for hypertension_ = 0.071, 95%CI: 0.058–0.086; OR_screening for diabetes mellitus_ = 0.098, 95%CI: 0.076–0.127; OR_influenza vaccination among < 60 with risk factor_ = 0.639, 95%CI: 0.357–1.143; OR_influenza vaccination among 60+_ = 0.330, 95%CI: 0.222–0.489). (Detailed models in **Appendix 3–6.**)

### Population estimations

According to estimations for the entire adult population of Hungary, there were 4.1 million interventions implemented per year (a total of 8.1 million interventions in the two investigated years) (Table 2), most of them (3.8 million/year) among adults who visited a GP in the previous year. The majority of each studied intervention was implemented among subjects who had visited a GP in the previous year (Table 3).

**Table 2:** Estimated numbers of implemented preventive actions per year in the entire population of Hungary in different target groups according to the observations of European Health Interview Surveys in 2009 and 2014

		number of implemented interventions	size of target population	proportion of implementation
blood pressure measurement^a^	EHIS2009	1,689,484	2,555,201	66.1%
	EHIS2014	1,795,068	2,640,770	68.0%
	both	3,484,552	5,195,971	67.1%
checking blood glucose^b^	EHIS2009	1,487,560	2,427,747	61.3%
	EHIS2014	1,590,200	2,422,453	65.6%
	both	3,077,760	4,850,200	63.5%
influenza vaccination, < 60 years^c^	EHIS2009	212,686	2,005,196	10.6%
	EHIS2014	137,541	1,509,136	9.1%
	both	350,227	3,514,332	10.0%
influenza vaccination, 60+ years^d^	EHIS2009	671,928	2,318,158	29.0%
	EHIS2014	553,324	2,430,944	22.8%
	both	1,225,252	4,749,102	25.8%
studied^a + b + c + d^ preventive services together	EHIS2009	4,061,658	9,306,302	43.6%
	EHIS2014	4,076,133	9,003,303	45.3%
	both	8,137,791	18,309,605	44.4%

**Table 3:** Number of adults from intervention-specific target populations in the two investigated years who used or did not use preventive services taking into consideration of the recommended intervention intervals in Hungary as a function of the timing of visiting a GP according to the European Health Interview Surveys of 2009 and 2014

		blood pressure measurement^a^	checking blood glucose^b^	influenza vaccination < 60^c^	influenza vaccination 60 ≤ ^d^	studied^a + b + c + d^ preventive services together
number of implemented interventions (proportion of recommended interventions)	GP visit in a year	3,072,445 (59.1%)	2,931,690 (79.9%)	328,181 (9.3%)	1,175,017 (24.7%)	7,507,333 (43.8%)
	no GP visit in a year	412,107 (7.9%)	146,070 (4%)	22,046 (0.6%)	50,235 (1.1%)	630,458 (3.7%)
	total	3,484,552 (67.1%)	3,077,760 (83.9%)	350,227 (10%)	1,225,252 (25.8%)	8,137,791 (47.5%)
number of missed interventions (proportion of recommended interventions)	GP visit in a year	561,098 (10.8%)	363,270 (9.9%)	2,784,072 (79.2%)	3,029,700 (63.8%)	6,738,140 (39.3%)
	no GP visit in a year	1,150,321 (22.1%)	227,543 (6.2%)	380,033 (10.8%)	494,150 (10.4%)	2,252,047 (13.1%)
	total	1,711,419 (32.9%)	590,813 (16.1%)	3,164,105 (90%)	3,523,850 (74.2%)	8,990,187 (52.5%)
number of recommended interventions	total	5,195,971 (100%)	3,668,573 (100%)	3,514,332 (100%)	4,749,102 (100%)	17,127,978 (100%)

The number of missed interventions taking into consideration the recommended screening intervals was 4.5 million per year. Most of them (3.4 million per year) were estimated for persons who had visited a GP in the previous year (Table 3).

The number of missed opportunities among subjects who visited a GP within the previous year varied by intervention. The number of missed opportunities was greatest for the influenza vaccination among the ≥60 HR group, followed by influenza vaccination among the < 60 group, hypertension screening, and diabetes mellitus screening.

The number of missed opportunities among persons who visited a GP more than a year prior was greatest for hypertension screening, followed by influenza vaccination among the ≥60 group, influenza vaccination among the < 60 HR group, and diabetes mellitus screening.

The opportunistic SAPI (among patients who visited a GP in the previous year) and organised SAPI (among patients who did not visit a GP in a year) for 2 years (with percentage of recommended interventions) were estimated to be 561,098 (10.8% of recommended interventions) and 1,150,321 (22.1%) for hypertension screening; 363,270 (9.9%) and 227,543 (6.2%) for diabetes mellitus screening; 2,784,072 (79.2%) and 380,033 (10.8%) for influenza vaccination among the < 60 HR group; and 3,029,700 (63.8%) and 494,150 (10.4%) for influenza vaccination among the ≥60 group.

### Estimation for an average-sized GMP

**Fig. 1:**
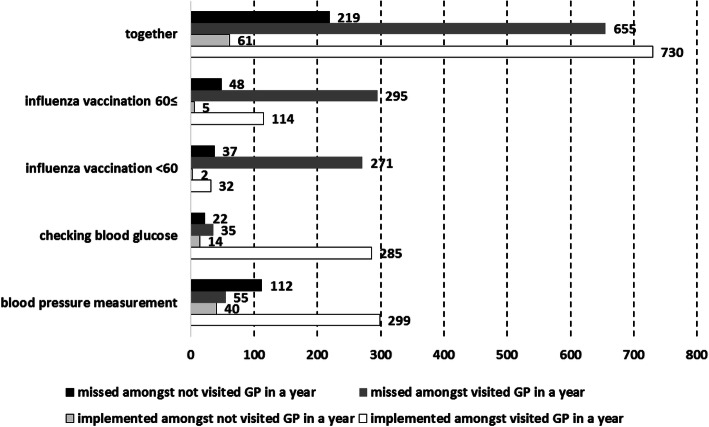
Number of adults from intervention-specific target populations in Hungary who used or did not use preventive services in the previous year in an average-sized general medical practice as a function of the timing of visiting a GP according to the European Health Interview Surveys of 2009 and 2014

The implemented and missed numbers of interventions per year in an average-sized Hungarian GMP were 791 and 874, respectively. The majority of missed (75.0%; 655/874) and utilised (92.3%; 730/791) opportunities estimated for subjects who had visited the GP in the previous year (Fig. 1).

Most of the implemented actions were hypertension screenings (299) followed by diabetes mellitus screenings (285). There were only 146 influenza vaccinations per year (114 among the ≥60 group and 32 among the < 60 HR group). The highest number of missed interventions among patients who visited the doctor in a year was observed for influenza vaccination (295 among the ≥60 group and 271 among the < 60 HR group), followed by cardio-metabolic screenings (55 hypertension screenings and 35 diabetes mellitus screenings).

The ratio of missed-to-implemented interventions among subjects who visited a GP in the previous year was highest for influenza vaccination (2.58 among the ≥60 group and 8.48 among the < 60 HR group). This ratio was 0.12 for diabetes mellitus screening and 0.18 for hypertension screening. Altogether, more actions were implemented than were missed (0.90). (Fig. 1).

The number of missed interventions among subjects who did not visit a GP in the previous year was 219 per year. The majority of them were missed blood pressure screenings (112), followed by missed influenza vaccination among the ≥60 group (48), influenza vaccination among the < 60 HR group (37), and blood glucose screening (22).

## Discussion

### Main findings

Our analysis demonstrated that screenings for hypertension and diabetes are much less intensive than recommended; and the implementation of influenza vaccination is critically neglected in Hungary. The proportion of subjects taken up hypertension screening in Hungary was similar to that from Poland [39] less than in Italy [40], and much less than reported in England, in Canada and in the United States [41–43]. The diabetes mellitus screening proportion in Denmark and in the UK was higher [44], in Poland was similar [39], and among Dutch with Asian origin [45] was much less than in Hungary. The Hungarian observations for influenza vaccination correspond to reports from Portugal [46]. The vaccination coverage was higher than the Hungarian in France [47], in Spain [48], and in the UK [49] in both studied target groups.

Hypertension screening had the highest implementation ratio, and it increased slightly between the survey years (66.1 to 68.0%). (Table 2) The lower estimate of the role of providers who were not GPs but who implemented the inventions was relatively high (7.9%). Because the 3,484,552 implemented screenings in 2 years were accompanied by 561,098 in 2 years opportunistic SAPI and 1,150,321 in 2 years organised SAPI (Table 3), the gain potentially achievable by improvement in screening implementation is larger for organised approaches.

Almost two-thirds of the target population underwent diabetes mellitus screening (Table 2). The lower approximation for the role of non-GPs is only 4.0%. The SAPI of the opportunistic approach (363,270/2 years) is remarkably higher than that of the organised approach (227,543/2 years) (Table 3).

The influenza vaccination for the < 60 HR group is an underused service in Hungary. Furthermore, the already very low implementation ratio showed a slight decrease between survey collections (10.6 to 9.1%) (Table 2). The role of GPs is not replaced by other physicians, as is reflected in the lower estimate of 0.6% for non-GP contributions. The highly negligent attitude of GPs is reflected in the larger opportunistic SAPI (2,784,072/2 years) compared to the organised SAPI (380,033/2 years) (Table 3).

The decreasing implementation ratio of influenza vaccination between data collection time points (29.0 to 22.8%) is very low but considerably higher among the < 60 group (Table 2). The role of non-GPs seems to be smaller than for the < 60 HR group. The opportunistic SAPI (3,029,700/2 years) is also much larger than the organised SAPI (494,150/2 years) for this vaccination target group (Table 3).

Altogether, the preventive interventions that can be delivered at the PHC level showed a small but slightly increased implementation ratio (43.6% vs 45.3%) (Table 2). The majority of the missed interventions (8,990,187/2 years) belong to the opportunistic SAPI (6,738,140/2 years). The organised SAPI is estimated to be 2,252,047/2 years. The role of GPs is not taken over by other physicians, as is reflected by the 3.7% lower estimation of non-GP contributions to the delivery of the investigated preventive interventions (Table 3).

### Implications

The unexploited SAPI of opportunistic prevention is much larger for influenza vaccination than for organised vaccination programmes. For diabetes screening, these SAPIs are similar. The organised SAPI is the dominant for hypertension screening.

As shown by our analysis, GPs delivered the majority of the investigated prevention services. More precisely, a minority of the interventions were delivered per year to adults who did not visit a GP in the previous year. Because it could not be excluded that an intervention was delivered by a non-GP physician, a lower approximation could be calculated in our analyses for the role of non-GPs. Since PHC service development is the key factor for both opportunistic and organised approaches, the workload of the GP determines the feasibility of any further development of PHC services.

At the time of the study period, 655 interventions were missed among patients of an average-sized GMP who visited GP in the previous year. This opportunistic SAPI corresponds to 12–13 extra interventions per week. The SAPI for organised approaches is 219 interventions a year (4–5 interventions per week). The whole SAPI is 16–18 extra interventions per week in an average-sized GMP.

The increase in the necessary workload is significant. Therefore, PHC service development requires a capacity increase in Hungarian GMPs. At present, a typical PHC staff consists of a GP (who is the owner of the GMP) and a nurse [50]. This minimal staff seems to be an obstacle for the development of effective preventive services irrespective of the nature of the preventive approach. Larger staffs with broader professional expertise are needed both for guideline-based opportunistic service delivery and for population-level, call-based organised service delivery [10].

### Strengths and limitations

The surveys on which our analysis is based were supervised by Eurostat with respect to the questionnaire content, sampling, and data collection. Eurostat’s involvement established the reliability of the investigation. The questionnaires used in the two surveys had the same questions on the parameters we investigated. Furthermore, the sample sizes from both surveys were large enough to ensure high statistical power.

The response rates of the surveys were not particularly high, jeopardizing the survey’s representativeness. This weakness was partly handled by weighting in statistical analyses, which took into consideration age, sex, and settlement type-specific response rates. All the estimations for the populations of the whole country and of the GMPs are approximations of numbers of interventions.

The morbidity status of participants was assessed by self-reporting. This could result in underreporting. Since screening interventions (measurement of blood pressure and checking glucose levels) are regularly applied as a part of chronic care for other than hypertension and diabetes mellitus, the proportion of screenings in the target groups could be overestimated. Consequently, the observed poor screening performance could be even worse.

The interventions among adults who visited a GP in the previous year were considered to be implemented by a GP. Even though subjects of our investigation were apparently healthy, few interventions could be implemented by non-GPs. This led to some overestimation of GP contribution to intervention delivery, but it did not influence the SAPI of organised prevention at PHC.

## Conclusions

The preventive intervention delivered by PHC providers has a poor implementation ratio in Hungary. Screenings for hypertension and diabetes mellitus are far less intensive than recommended. The influenza vaccination rate among adults is especially critical.

The opportunistic SAPI is dominant in the case of influenza vaccination. The implementation ratio of hypertension screening could be improved by an organised approach. The opportunistic and organised screenings’ SAPIs are similar in the case of diabetes screening.

Considering the prerequisites for organised and opportunistic interventions, opportunistic approaches should be prioritised in short-term policy formulation. SAPI of the organised approach could be exploited if the GMP team could be enhanced with new professionals who could operate a population-based organisation.

## Data Availability

The datasets used and/or analysed during the current study are available from the corresponding author on reasonable request. The database of original surveys is not available publically. The project of the data processing in order to publish a paper was initiated by the institution responsible for the Hungarian implementation of European Health Interview Surveys, by the Hungarian Central Statistical Office. Authors’ was invited for the secondary analysis and for the manuscript preparation.

## Appendix 1

List of diseases used in assessment of high risk status for influenza vaccination among adults less than 60 years old

### asthma

- chronic bronchitis
- emphysema
- ischemic heart disease

### angina

- hypertension
- diabetes mellitus
- chronic liver disease
- liver cirrhosis
- malignant disease
- lipid disorder

### arrhythmia

- atrial flutter
- any other chronic heart disorder
- former myocardial infraction
- former stroke, and transient ischemic attack

## Appendix 2

List of chronic disorders specified by responders of European Health Interview Surveys if they reported the presence of at least one chronic disorder

- asthma (allergic asthma included)
- chronic bronchitis, chronic obstructive pulmonary disease, emphysema
- myocardial infraction
- coronary heart disease and angina
- hypertension
- stroke

### arrhythmia

- other heart disease
- rheumatic disorder
- arthrosis and osteoarthritis
- low back disorder or other chronic back defect
- neck disorder or other chronic neck defect
- osteoporosis
- diabetes mellitus
- dyslipidemia
- allergy except allergic asthma
- gastric or duodenal ulcer
- chronic liver disease
- malignant disease
- urinary incontinence, problems in controlling the bladder chronic renal disease
- severe headache, migraine
- chronic anxiety
- chronic depression
- other mental health problems
- chronic disability by trauma

## Appendix 3

**Table 4:** Factors influencing the implementation of blood pressure measurement in a year among adults at least 40 years old and free of diagnosed hypertension in Hungary according to the European Health Interview Surveys in 2009 and 2014 by multivariate logistic regression analysis

Factor	OR [95%CI]	***P***-value
sex (female/male)	0.97 [0.78;1.2]	0.77
age group (45–49/40–44)	0.78 [0.59;1.04]	0.097
age group (50–54/40–44)	0.72 [0.53;0.97]	0.03
age group (55–59/40–44)	0.8 [0.58;1.12]	0.192
age group (60–64/40–44)	0.62 [0.43;0.87]	0.006
age group (65–69/40–44)	0.48 [0.32;0.71]	< 0.001
age group (70–74/40–44)	0.63 [0.39;1.02]	0.061
age group (75–79/40–44)	1.28 [0.71;2.32]	0.412
age group (80–84/40–44)	1.36 [0.67;2.77]	0.392
age group (85+/40–44)	1.16 [0.52;2.55]	0.72
family status (single/married, living together)	0.71 [0.52;0.98]	0.037
family status (married, living separated/married, living together)	0.57 [0.33;0.99]	0.046
family status (divorced/married, living together)	0.92 [0.71;1.2]	0.551
family status (widow/married, living together)	0.88 [0.63;1.23]	0.446
education (vocational/primary)	0.97 [0.74;1.27]	0.82
education (high-school/primary)	1.4 [1.05;1.86]	0.023
education (tertiary/primary)	1.55 [1.14;2.1]	0.005
alcohol (occasional/never)	1.31 [1.05;1.63]	0.017
alcohol (moderate/never)	1.42 [1.04;1.95]	0.029
alcohol (regular/never)	1.55 [1.1;2.18]	0.013
alcohol (heavy/never)	0.88 [0.51;1.51]	0.641
BMI (thin/normal)	0.83 [0.55;1.26]	0.385
BMI (overweighed/normal)	0.95 [0.77;1.17]	0.61
BMI (obese/normal)	1.22 [0.93;1.6]	0.161
smoking (ceased/never)	1.08 [0.84;1.39]	0.532
smoking (moderate/never)	0.85 [0.66;1.09]	0.195
smoking (heavy/never)	0.6 [0.44;0.82]	0.001
smoking (other/never)	1.16 [0.57;2.36]	0.68
general health (very bad/fair)	1.81 [0.98;3.36]	0.06
general health (bad/fair)	1.85 [1.25;2.74]	0.002
general health (good/fair)	0.93 [0.74;1.17]	0.549
general health (very good/fair)	0.94 [0.68;1.3]	0.716
chronic disease (present/absent)	1.58 [1.26;1.98]	< 0.001
visiting GP (more than a year/in a year)	0.07 [0.06;0.09]	< 0.001
EHIS wave (EHIS2014/EHIS2009)	1.4 [1.14;1.71]	0.001

## Appendix 4

**Table 5:** Factors influencing the implementation of blood glucose checking in a year among adults at least 45 years old, overweighed or obese, and free of diagnosed diabetes mellitus in Hungary according to the European Health Interview Surveys in 2009 and 2014 by multivariate logistic regression analysis

Factor	OR [95%CI]	***P***-value
sex (female/male)	1 [0.82;1.23]	0.99
age group (50–54/45–49)	1.22 [0.9;1.66]	0.199
age group (55–59/45–49)	1.1 [0.81;1.49]	0.546
age group (60–64/45–49)	0.99 [0.73;1.35]	0.94
age group (65–69/45–49)	1.09 [0.77;1.52]	0.635
age group (70–74/45–49)	1.26 [0.86;1.82]	0.232
age group (75–79/45–49)	1.31 [0.86;2]	0.21
age group (80–84/45–49)	1.23 [0.74;2.04]	0.429
age group (85+/45–49)	1.47 [0.76;2.86]	0.252
family status (single/married, living together)	0.65 [0.45;0.95]	0.028
family status (married, living separated/married, living together)	0.75 [0.38;1.47]	0.398
family status (divorced/married, living together)	1.19 [0.9;1.58]	0.214
family status (widow/married, living together)	0.98 [0.77;1.26]	0.896
education (vocational/primary)	1.77 [1.39;2.26]	< 0.001
education (high-school/primary)	2.13 [1.64;2.76]	< 0.001
education (tertiary/primary)	2.35 [1.77;3.12]	< 0.001
alcohol (occasional/never)	1.13 [0.92;1.39]	0.238
alcohol (moderate/never)	1.17 [0.87;1.58]	0.295
alcohol (regular/never)	0.91 [0.67;1.23]	0.535
alcohol (heavy/never)	0.84 [0.47;1.48]	0.539
BMI (obese/ overweighed)	1.01 [0.85;1.2]	0.922
smoking (ceased/never)	1.12 [0.91;1.39]	0.283
smoking (moderate/never)	0.88 [0.67;1.15]	0.347
smoking (heavy/never)	0.66 [0.46;0.94]	0.02
smoking (other/never)	1.11 [0.53;2.34]	0.777
general health (very bad/fair)	2.14 [1.34;3.43]	0.002
general health (bad/fair)	1.8 [1.36;2.38]	< 0.001
general health (good/fair)	0.95 [0.77;1.17]	0.646
general health (very good/fair)	0.71 [0.48;1.05]	0.083
chronic disease (present/absent)	2.3 [1.84;2.88]	< 0.001
visiting GP (more than a year/in a year)	0.1 [0.08;0.13]	< 0.001
EHIS wave (EHIS2014/EHIS2009)	1.71 [1.42;2.05]	< 0.001

## Appendix 5

**Table 6:** Factors influencing the implementation of vaccination against seasonal influenza epidemic among adults less than 60 years old with diagnosed chronic disease* in Hungary according to the European Health Interview Surveys in 2009 and 2014 by multivariate logistic regression analysis

	INF60-	
**Factor**	**OR [95%CI]**	***P*****-value**
sex (female/male)	0.93 [0.67;1.29]	0.652
age group (25–29/20–24)	0.18 [0.04;0.85]	0.031
age group (30–34/20–24)	0.59 [0.22;1.57]	0.29
age group (35–39/20–24)	0.86 [0.37;2]	0.718
age group (40–44/20–24)	0.99 [0.42;2.33]	0.99
age group (45–49/20–24)	0.98 [0.42;2.28]	0.954
age group (50–54/20–24)	0.86 [0.38;1.99]	0.73
age group (55–59/20–24)	1.24 [0.54;2.82]	0.609
family status (single/married, living together)	1.19 [0.76;1.87]	0.445
family status (married, living separated/married, living together)	1.07 [0.37;3.12]	0.904
family status (divorced/married, living together)	0.82 [0.52;1.29]	0.383
family status (widow/married, living together)	0.93 [0.49;1.77]	0.836
education (vocational/primary)	1.36 [0.85;2.15]	0.196
education (high-school/primary)	2.25 [1.42;3.58]	0.001
education (tertiary/primary)	2.41 [1.44;4.02]	0.001
alcohol (occasional/never)	0.98 [0.7;1.37]	0.897
alcohol (moderate/never)	0.53 [0.29;0.96]	0.037
alcohol (regular/never)	1.14 [0.63;2.07]	0.654
alcohol (heavy/never)	1.35 [0.56;3.26]	0.506
BMI (thin/normal)	1.08 [0.56;2.12]	0.813
BMI (overweighed/normal)	0.93 [0.63;1.38]	0.719
BMI (obese/normal)	1.16 [0.79;1.72]	0.455
smoking (ceased/never)	1.45 [1.02;2.06]	0.037
smoking (moderate/never)	0.74 [0.48;1.12]	0.153
smoking (heavy/never)	0.51 [0.28;0.94]	0.031
smoking (other/never)	1.19 [0.48;2.93]	0.711
general health (very bad/fair)	1.88 [0.99;3.57]	0.055
general health (bad/fair)	1.96 [1.32;2.92]	0.001
general health (good/fair)	0.78 [0.54;1.12]	0.184
general health (very good/fair)	0.77 [0.36;1.65]	0.508
chronic disease (present/absent)	0.87 [0.52;1.45]	0.601
visiting GP (more than a year/in a year)	0.64 [0.36;1.14]	0.131
EHIS wave (EHIS2014/EHIS2009)	0.86 [0.63;1.17]	0.342

*hypertension, ischaemic heart disease, myocardial infraction, stroke, arrhythmia, heart failure, chronic respiratory disease, asthma, diabetes mellitus, dyslipidaemia, chronic liver disease, cancer

## Appendix 6

**Table 7:** Factors influencing the implementation of vaccination against seasonal influenza epidemic among adults at least 60 years old in Hungary according to the European Health Interview Surveys in 2009 and 2014 by multivariate logistic regression analysis

Factor	OR [95%CI]	*P*-value
sex (female/male)	1.06 [0.86;1.32]	0.579
age group (65–69/60–64)	1.72 [1.33;2.21]	< 0.001
age group (70–74/60–64)	1.94 [1.48;2.53]	< 0.001
age group (75–79/60–64)	2.03 [1.52;2.71]	< 0.001
age group (80–84/60–64)	2.38 [1.69;3.34]	< 0.001
age group (85+/60–64)	2.35 [1.57;3.51]	< 0.001
family status (single/married, living together)	1.06 [0.62;1.79]	0.836
family status (married, living separated/married, living together)	0.66 [0.28;1.54]	0.337
family status (divorced/married, living together)	0.63 [0.44;0.88]	0.008
family status (widow/married, living together)	0.82 [0.66;1.01]	0.068
education (vocational/primary)	1.04 [0.81;1.33]	0.765
education (high-school/primary)	1.43 [1.12;1.84]	0.005
education (tertiary/primary)	1.84 [1.42;2.38]	< 0.001
alcohol (occasional/never)	1.05 [0.85;1.29]	0.638
alcohol (moderate/never)	1 [0.72;1.37]	0.982
alcohol (regular/never)	1.22 [0.92;1.61]	0.172
alcohol (heavy/never)	0.68 [0.36;1.29]	0.235
BMI (thin/normal)	0.55 [0.32;0.94]	0.029
BMI (overweighed/normal)	0.91 [0.74;1.12]	0.378
BMI (obese/normal)	1 [0.79;1.26]	0.995
smoking (ceased/never)	1.1 [0.89;1.36]	0.381
smoking (moderate/never)	0.7 [0.5;0.99]	0.042
smoking (heavy/never)	0.8 [0.49;1.31]	0.382
smoking (other/never)	0.2 [0.05;0.87]	0.032
general health (very bad/fair)	0.88 [0.65;1.2]	0.432
general health (bad/fair)	1.03 [0.82;1.28]	0.816
general health (good/fair)	0.76 [0.59;0.97]	0.027
general health (very good/fair)	0.95 [0.53;1.7]	0.854
chronic disease (present/absent)	2.13 [1.53;2.98]	< 0.001
visiting GP (more than a year/in a year)	0.33 [0.22;0.49]	< 0.001
EHIS wave (EHIS2014/EHIS2009)	0.72 [0.61;0.86]	< 0.001

## Abbreviations

PHC: Primary health care
GP: General practitioner
EHIS2009, EHIS2014: European Health Interview Surveys collected in 2009 and 2014
USPSTF: U.S. Preventive Services Task Force
CDC: Centers for Disease Control and Prevention
NHS: National Health Service
≥60: Persons at least 60 years old
< 60 HR: Persons 20–59 years old with one of the high-risk diseases
GMP: General medical practice
SAPI: Number of potential subjects available for preventive intervention by GPs
non-GP: Another physician not a GP
OR: Odds ratios
95% CI: 95% confidence intervals

## References

[1] WHO (1978). Declaration of Alma Ata. International conference on primary health care, Alma-Ata, USSR.

[2] Atun R (2004). What are the advantages and disadvantages of restructuring a health care system to be more focused on primary care services? Health evidence network.

[3] Davletov K, Nurgozhin T, McKee M (2018). Reflecting on Alma Ata 1978: forty years on. Eur J Pub Health.

[4] Maeseneer J, Kendall S (2018). Primary health care 40 years after Alma Ata 1978: addressing new challenges in a changing society. Eur J Pub Health.

[5] Gillam S (2008). Is the declaration of Alma Ata still relevant to primary health care?. BMJ..

[6] Field K, Thorogood M, Silagy C, Normand C, O'Neill C, Muir J (1995). Strategies for reducing coronary risk factors in primary care: which is most cost effective?. BMJ..

[7] Wonderling D, McDermott C, Buxton M, Kinmonth AL, Pyke S, Thompson S, Wood D (1996). Costs and cost effectiveness of cardiovascular screening and intervention: the British family heart study. BMJ..

[8] Recommendations for Primary Care Practice (2017). U.S. Preventive Services Task Force.

[9] The Royal Australian College of General Practitioners (2016). Guidelines for preventive activities in general practice.

[10] Expert Panel on Effective Ways of Investing in Health. Report on Definition of a Frame of Reference in Relation to Primary Care with a Special Emphasis on Financing Systems and Referral Systems: Brussels; 2014. http://ec.europa.eu/health/expert_panel/sites/expertpanel/files/004_definitionprimarycare_en.pdf Accessed 28 Mar 2019.

[11] Unverzagt S, Oemler M, Braun K, Klement A (2014). Strategies for guideline implementation in primary care focusing on patients with cardiovascular disease: a systematic review. Fam Pract.

[12] Shanbhag D, Graham ID, Harlos K, Haynes RB, Gabizon I, Connolly SJ, Van Spall HGC. Effectiveness of implementation interventions in improving physician adherence to guideline recommendations in heart failure: a systematic review. BMJ Open. 2018. 10.1136/bmjopen-2017-017765. 10.1136/bmjopen-2017-017765 PMC5855256 29511005

[13] Jeffery RA, To MJ, Hayduk-Costa G, Cameron A, Taylor C, Van Zoost C, Hayden JA. Interventions to improve adherence to cardiovascular disease guidelines: a systematic review. BMC Fam Pract. 2015. 10.1186/s12875-015-0341-7. 10.1186/s12875-015-0341-7 PMC4619086 26494597

[14] Magnussen L, Ehiri J, Jolly P. Comprehensive versus selective primary health care: lessons for global health policy. Health Aff. 2004. 10.1377/hlthaff.23.3.167. 10.1377/hlthaff.23.3.167 15160814

[15] Krogsbøll LT, Jørgensen KJ, Larsen CG, Gøtzsche PC (2012). General health checks in adults for reducing morbidity and mortality from disease: Cochrane systematic review and meta-analysis. BMJ..

[16] Jørgensen T, Jacobsen RK, Toft U, Aadahl M, Glümer C, Pisinger C (2014). Effect of screening and lifestyle counselling on incidence of ischaemic heart disease in general population: Inter99 randomised trial. BMJ..

[17] ESCAP (2014). Inter99 trial: a statement from the NHS Health Check Expert Scientific and Clinical Advisory Panel.

[18] Sándor J, Kósa K, Fürjes G, Papp M, Csordás Á, Rurik I, Ádány R (2013). Public health services provided in the framework of general practitioners’ clusters. Eur J Pub Health.

[19] Sándor J, Nagy A, Földvári A, Szabó E, Csenteri O, Vincze F, Sipos V, Kovács N, Pálinkás A, Papp M, Fürjes G, Ádány R (2017). Delivery of cardio-metabolic preventive services to Hungarian Roma of different socio-economic strata. Fam Pract.

[20] Sándor J, Kósa K, Papp M, Fürjes G, Kőrösi L, Jakovljevic M, Ádány R (2016). Capitation-based financing hampers the provision of preventive Services in Primary Health Care. Front Public Health.

[21] Sándor J, Nagy A, Jenei T, Földvári A, Szabó E, Csenteri O, Vincze F, Sipos V, Kovács N, Pálinkás A, Papp M, Fürjes G, Ádány R (2018). Influence of patient characteristics on preventive service delivery and general practitioners' preventive performance indicators: a study in patients with hypertension or diabetes mellitus from Hungary. Eur J Gen Pract.

[22] Regulation (EC) No 1338/2008 of the European Parliament and of the Council of 16 December 2008 on Community statistics on public health and health and safety at work. https://eur-lex.europa.eu/eli/reg/2008/1338/oj Accessed 28 Mar 2019.

[23] Commission Regulation (EU) No 141/2013 of 19 February 2013 implementing Regulation (EC) No 1338/2008 of the European Parliament and of the Council on Community statistics on public health and health and safety at work, as regards statistics based on the European Health Interview Survey (EHIS) https://eur-lex.europa.eu/eli/reg/2013/141/oj Accessed 28 Mar 2019.

[24] EUROSTAT: EHIS wave 1 guidelines. European Commission, Directorate F: Social Statistics and Information Society. https://ec.europa.eu/eurostat/web/microdata/european-health-interview-survey Accessed 16 June 2020.

[25] EUROSTAT: EHIS wave 2 variables. https://ec.europa.eu/eurostat/web/microdata/european-health-interview-survey Accessed 16 June 2020.

[26] Hungarian Central Statistical Office. European Health Interview Survey. http://www.ksh.hu/elef/archiv/2009/celok.html [in Hungarian] Accessed 28 Mar 2019.

[27] Hungarian Central Statistical Office. European Health Interview Survey. http://www.ksh.hu/elef/celok.html [in Hungarian] Accessed 28 Mar 2019.

[28] U.S (2007). Preventive Services Task Force: Screening for High Blood Pressure: U.S. Preventive Services Task Force Reaffirmation Recommendation Statement. Ann Intern Med.

[29] Piper MA, Evans CV, Burda BU, Margolis KL, O'Connor E, Smith N, Webber E, Perdue LA, Bigler KD, Whitlock EP (2014). Screening for high blood pressure in adults: a systematic evidence review for the U.S. preventive services task force. Evidence synthesis no. 121. AHRQ publication no. 13–05194-EF-1.

[30] Hungarian Society on Hypertension (2009). Recommendation on the care for hypertension among children and adults. [in Hungarian].

[31] American Diabetes Association (2007). Standards of medical care in diabetes. Diabetes Care.

[32] U.S (2008). Preventive Services Task Force. Screening for Type 2 Diabetes Mellitus in Adults: U.S. Preventive Services Task Force Recommendation Statement. Ann Intern Med.

[33] Board of Hungarian General Practitioners (2006). Guideline on the care of diabetes mellitus among adults by general practitioner. [in Hungarian].

[34] Salisbury D, Ramsay M, Noakes K (2006). Immunisation against infectious disease.

[35] Csohán Á, Molnár Z, Jelenik Z, Melles M, Pauliny Z, Békési Z (2009). Guideline of the Hungarian Epidemiological Center on vaccination in 2009 [in Hungarian].

[36] Vik PW, Culbertson KA, Sellers K (2000). Readiness to change drinking among heavy-drinking college students. J Stud Alcohol.

[37] WHO (1995). Physical status: the use and interpretation of anthropometry. Report of a WHO Expert Committee. WHO Technical Report Series 854.

[38] Eurostat (2013). European Health Interview Survey (EHIS wave 2) Methodological manual.

[39] Gowin E, Avonts D, Horst-Sikorska W, Dytfeld J, Michalak M (2012). Stimulating preventive procedures in primary care: Effect of PIUPOZ program on the delivery of preventive procedures. Arch Med Sci.

[40] Manuti B, Rizza P, Bianco A, Nobile CGA, Pavia M (2010). The Quality of Preventive Health Care Delivered to Adults: Results From a Cross-Sectional Study in Southern Italy. BMC Public Health.

[41] Dalton ARH, Bottle A, Okoro C, Majeed A, Millett C (2011). Implementation of the NHS Health Checks programme: baseline assessment of risk factor recording in an urban culturally diverse setting. Fam Pract.

[42] Dubey V, Mathew R, Iglar K, Moineddin R, Glazier R (2006). Improving preventive service delivery at adult complete health check-ups: the Preventive health Evidence-based Recommendation Form (PERFORM) cluster randomized controlled trial. BMC Fam Pract.

[43] Shires DA, Stange KC, Divine G, Ratliff S, Vashi R, Tai-Seale M (2012). Jennifer Elston Lafata: prioritization of evidence-based preventive health services during periodic health examinations. Am J Prev Med.

[44] van den Donk M, Sandbaek A, Borch-Johnsen K, Lauritzen T, Simmons RK, Wareham NJ, Griffin SJ, Davies MJ, Khunti K, Rutten GEHM (2011). Screening for type 2 diabetes: lessons from the ADDITION-Europe study. Diabet Med.

[45] van Valkengoed IGM, Vlaar EMA, Nierkens V, Middelkoop BJC, Stronks K. The Uptake of Screening for Type 2 Diabetes and Prediabetes by Means of Glycated Hemoglobin versus the Oral Glucose Tolerance Test among 18 to 60-Year-Old People of South Asian Origin: A Comparative Study. PLoS One. 10(8):e0136734. 10.1371/journal pone.0136734. 10.1371/journal.pone.0136734 PMC4552828 26317417

[46] Pinto CS, Nunes B, Branco MJ, Falcão JM (2013). Trends in influenza vaccination coverage in Portugal from 1998 to 2010: effect of major pandemic threats. BMC Public Health.

[47] Verger P, Fressard L, Cortaredona S, Lévy-Bruhl D, Loulergue P, Galtier F, Bocquier A (2018). Trends in seasonal influenza vaccine coverage of target groups in France, 2006/07 to 2015/16: Impact of recommendations and 2009 influenza A(H1N1) pandemic. Euro Surveill.

[48] Rosell-Murphy M, Rodriguez-Blanco T, Morán J, Pons-Vigués M, Elorza-Ricart JM, Rodríguez J, Pareja C, Nuin MÁ, Bolíbar B (2015). Variability in screening prevention activities in primary care in Spain: a multilevel analysis. BMC Public Health.

[49] Müller D, Nguyen-Van-Tam JS, Szucs TD (2006). Influenza vaccination coverage rates in the UK: a comparison of two monitoring methods during the 2002–2003 and 2003–2004 seasons. Public Health.

[50] Gaál P, Szigeti S, Csere M, Gaskins M, Panteli D (2011). Hungary: health system review. Health Syst Transit.

[51] Act CLV of 2016 on Official Statistics [2016. évi CLV. törvény a hivatalos statisztikáról, in Hungarian] https://net.jogtar.hu/jogszabaly?docid=a1600155.tv Accessed 16 June 2020.

[52] Government Decree 184/2017 [184/2017. (VII. 5.) Korm. rendelet a hivatalos statisztikáról szóló 2016. évi CLV. törvény végrehajtásáról; in Hungarian] https://net.jogtar.hu/jogszabaly?docid=A1700184.KOR Accessed 16 June 2020.

